# Exploring the association between patient satisfaction and hospital quality in the United States

**DOI:** 10.1371/journal.pone.0352756

**Published:** 2026-07-07

**Authors:** Sora Kang, Huazhi Liu, Charles Ledonio, Darwin Ang

**Affiliations:** 1 Florida State University College of Medicine, Tallahassee, Florida, United States of America; 2 HCA Florida Ocala Hospital, Ocala, Florida, United States of America; 3 University of Central Florida, Orlando, Florida, United States of America; University of California Los Angeles, UNITED STATES OF AMERICA

## Abstract

**Background:**

Patient experience is increasingly used as a public quality metric, but its relationship to hospital safety grades remains incompletely characterized because patient reported experience and technical safety measures capture different dimensions of care.

**Methods:**

We performed a retrospective cross-sectional analysis of publicly available hospital level data from 2023. Leapfrog Hospital Safety Grades were linked with Hospital Consumer Assessment of Healthcare Providers and Systems (HCAHPS) patient experience scores. The linked dataset included 1,442 U.S. hospitals; 1,390 hospitals with letter grades A through F were included in grade stratified and regression analyses. HCAHPS domains were analyzed on a 1–5 scale, with higher scores indicating more favorable patient reported experience. One way ANOVA compared mean HCAHPS scores across Leapfrog grades. Multivariable logistic regression with hospital size adjustment evaluated HCAHPS domains associated with Leapfrog Grade A versus grades B through F.

**Results:**

Mean HCAHPS domain scores declined across most domains as Leapfrog grades worsened from A to F. In multivariable analysis, nurse communication was associated with higher odds of receiving a Grade A safety rating (odds ratio [OR] 1.54, 95% confidence interval [CI] 1.11 to 2.13; p = 0.01), as was the hospital recommendation score (OR 1.54, 95% CI 1.15 to 2.06; p = 0.004). Doctor communication showed an inverse conditional association (OR 0.75, 95% CI 0.59 to 0.97; p = 0.03). Other HCAHPS domains were not independently associated with Grade A after adjustment.

**Conclusions:**

Hospitals with higher Leapfrog safety grades generally had more favorable patient reported experience scores, and nurse communication and hospital recommendation were the most clinically interpretable independent predictors of Grade A status. The inverse association for doctor communication should be interpreted cautiously because HCAHPS domains are correlated and the analysis used hospital level cross-sectional data. These findings support the complementary role of patient experience in hospital quality assessment but do not establish causality.

## Introduction

Healthcare quality is commonly understood as the delivery of safe, effective, timely, efficient, equitable, and patient centered care. In the United States, these dimensions are measured through a fragmented ecosystem of public and private quality programs, each emphasizing different aspects of hospital performance [1,2]. Patient centered care has therefore become an important component of quality measurement because it captures domains of care that may not be reflected by mortality, readmission, infection, or process measures alone [3].

HCAHPS provides a national, standardized, publicly reported measure of hospitalized patients’ experiences, including communication with nurses and physicians, staff responsiveness, communication about medications, discharge information, care transitions, cleanliness, quietness, overall hospital rating, and willingness to recommend the hospital. Prior national work has shown that hospitals can provide both strong clinical care and favorable patient experience, and systematic reviews have found positive associations between patient experience, patient safety, and clinical effectiveness across multiple settings [4,5]. Other studies have found that patient experience surveys can complement technical quality measures, but the magnitude and consistency of these relationships vary by outcome, hospital type, patient population, and measurement strategy [6–10].

Hospital safety ratings are also heterogeneous. The Leapfrog Hospital Safety Grade was developed as a composite patient safety score using multiple safety indicators, but public hospital ratings systems often disagree because they use different measures, weights, data sources, and inclusion criteria [11,12]. In addition, studies of Leapfrog related measures have raised concerns about voluntary reporting, self-reported components, and imperfect alignment with other publicly reported quality measures [13,14]. These concerns do not make Leapfrog grades invalid; rather, they underscore that Leapfrog safety grades and HCAHPS patient experience scores are related but nonidentical indicators of hospital performance.

The current literature therefore leaves a practical question for hospital leaders and quality teams. Specifically, which HCAHPS domains are most closely associated with top tier hospital safety grades when multiple patient experience domains are considered simultaneously? Prior work has emphasized the importance of communication, particularly nursing and physician communication, but studies vary in their adjustment strategies and quality endpoints [15–18]. Understanding these associations may help identify patient experience domains that are most aligned with broader safety and quality performance while also clarifying the limitations of using patient satisfaction as a proxy for clinical quality.

The objective of this study was to evaluate the association between HCAHPS patient experience domains and Leapfrog Hospital Safety Grades in a national sample of U.S. hospitals. We hypothesized that higher patient experience scores would be associated with higher Leapfrog safety grades, but that individual HCAHPS domains would differ in their independent associations after adjustment for hospital size.

## Methods

### Study design and data sources

This was a retrospective cross-sectional study using publicly available, de-identified, hospital level data from 2023. Leapfrog Hospital Safety Grades were linked with HCAHPS patient experience measures. Because all data were publicly available and de-identified at the hospital level, no patient level information was accessed and institutional review board review was not required under our institutional policy.

The linked dataset included 1,442 U.S. hospitals. Leapfrog grades were distributed as follows: A, 407 hospitals; B, 312 hospitals; C, 573 hospitals; D, 91 hospitals; F, 7 hospitals; no grade, 11 hospitals; and not available, 41 hospitals. Hospitals with no grade or not available Leapfrog status were included only in descriptive data source accounting and were excluded from grade stratified and regression analyses. The final analytic sample for comparisons across A through F grades was therefore 1,390 hospitals.

### Variables and measures

The primary hospital quality outcome was Leapfrog Grade A versus all other graded hospitals, B through F. This dichotomization was selected because Grade A represents a readily interpretable top tier safety benchmark for consumers, quality leaders, and hospital administrators. We also reported the full grade distribution to make the implications of this dichotomization transparent. HCAHPS domains were analyzed as hospital level 1–5 scores, with higher values indicating more favorable patient-reported experience. Domains included overall hospital rating, nurse communication, doctor communication, staff responsiveness, communication about medicines, discharge information, care transition, cleanliness, quietness, and recommend hospital. Hospital size, measured as staffed beds, was included as an adjustment variable.

### Statistical analysis

Descriptive statistics summarized hospital grade distribution and HCAHPS domain scores. Mean HCAHPS scores were compared across Leapfrog grade categories using one-way ANOVA; the associated F-test evaluated whether mean HCAHPS scores differed across more than two grade groups. Because Leapfrog grade is categorical/ordinal, descriptive figures were not used to generate trendline derived R-squared values. Multivariable logistic regression was used to estimate the association between HCAHPS domains and Leapfrog Grade A status. Odds ratios, 95% confidence intervals, and p values were reported. The model included all HCAHPS domains and staffed beds. Statistical significance was defined as p < 0.05. Because the HCAHPS domains are conceptually and statistically correlated, model coefficients were interpreted as conditional associations rather than causal effects.

## Results

The linked dataset included 1,442 hospitals, of which 1,390 had Leapfrog grades A through F and were included in grade stratified and regression analyses. Among graded hospitals, 407 (29.3%) had Grade A, 312 (22.4%) had Grade B, 573 (41.2%) had Grade C, 91 (6.5%) had Grade D, and 7 (0.5%) had Grade F.

**Fig 1:**
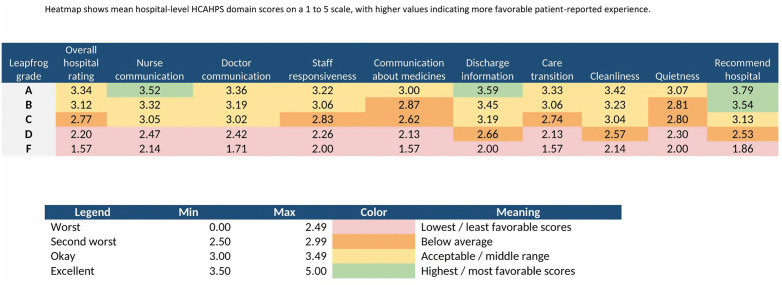
Mean HCAHPS domain scores by Leapfrog Hospital Safety Grade. Heatmap shows hospital level mean HCAHPS scores on a 1 to 5 scale, with higher values indicating more favorable patient reported experience. No trendline derived R-squared values are shown because Leapfrog grade is categorical/ordinal.

Across nearly all HCAHPS domains, mean scores decreased as Leapfrog grades worsened from A to F (Table 1; Fig 1). For example, the mean overall hospital rating was 3.337 among Grade A hospitals, 3.119 among Grade B hospitals, 2.770 among Grade C hospitals, 2.198 among Grade D hospitals, and 1.571 among Grade F hospitals. Similarly, mean nurse communication scores decreased from 3.518 among Grade A hospitals to 2.143 among Grade F hospitals, and recommend hospital scores decreased from 3.794 to 1.857.

**Table 1:** Mean HCAHPS domain scores by Leapfrog Hospital Safety Grade.

HCAHPS domain	A (n = 407)	B (n = 312)	C (n = 573)	D (n = 91)	F (n = 7)	ANOVA p value
Overall hospital rating	3.337	3.119	2.770	2.198	1.571	0.0032
Nurse communication	3.518	3.317	3.051	2.473	2.143	0.0024
Doctor communication	3.359	3.192	3.016	2.418	1.714	0.0123
Staff responsiveness	3.219	3.061	2.832	2.264	2	0.0039
Communication about medicines	3	2.872	2.620	2.132	1.571	0.0070
Discharge information	3.587	3.452	3.188	2.659	2	0.0086
Care transition	3.332	3.064	2.738	2.132	1.571	0.0021
Cleanliness	3.418	3.234	3.040	2.571	2.143	0.0039
Quietness	3.066	2.811	2.801	2.297	2	0.0082
Recommend hospital	3.794	3.542	3.126	2.527	1.857	0.0023

**Fig 2:**
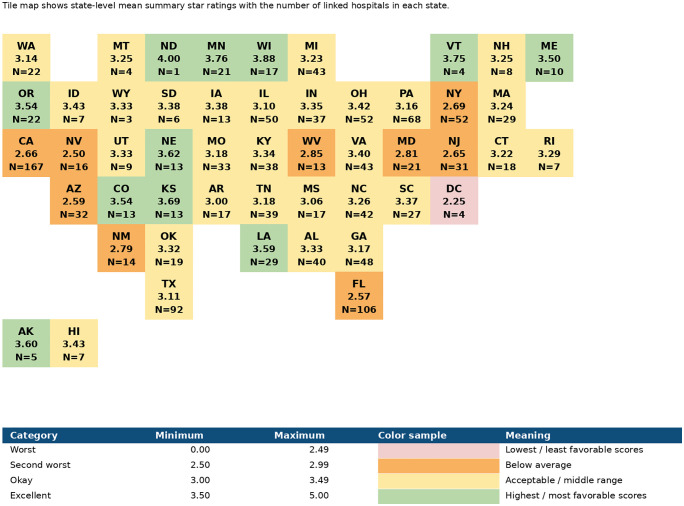
State level variation in mean HCAHPS summary star rating. Tile map shows state level mean summary star ratings with the number of hospitals in each state shown within the tile. Values should be interpreted descriptively because state sample sizes varied and several states had small numbers of linked hospitals.

State level HCAHPS summary star ratings varied across the analytic dataset (Table 2; Fig 2). Because state sample sizes differed substantially, including small numbers of hospitals in several states, these state level values were interpreted descriptively and were not used as stand alone evidence of state quality differences.

**Table 2:** State-level distribution of HCAHPS summary star ratings in the linked dataset.

State	# hospitals	Mean HCAHPS summary star rating
AK	5	3.6
AL	40	3.325
AR	17	3
AZ	32	2.594
CA	167	2.659
CO	13	3.538
CT	18	3.222
DC	4	2.25
FL	106	2.566
GA	48	3.167
HI	7	3.429
IA	13	3.385
ID	7	3.429
IL	50	3.1
IN	37	3.351
KS	13	3.692
KY	38	3.342
LA	29	3.586
MA	29	3.241
MD	21	2.81
ME	10	3.5
MI	43	3.233
MN	21	3.762
MO	33	3.182
MS	17	3.059
MT	4	3.25
NC	42	3.262
ND	1	4
NE	13	3.615
NH	8	3.25
NJ	31	2.645
NM	14	2.786
NV	16	2.5
NY	52	2.692
OH	52	3.423
OK	19	3.316
OR	22	3.545
PA	68	3.162
RI	7	3.286
SC	27	3.37
SD	6	3.383
TN	39	3.179
TX	92	3.109
UT	9	3.333
VA	43	3.395
VT	4	3.75
WA	22	3.136
WI	17	3.882
WV	13	2.846
WY	3	3.333

**Fig 3:**
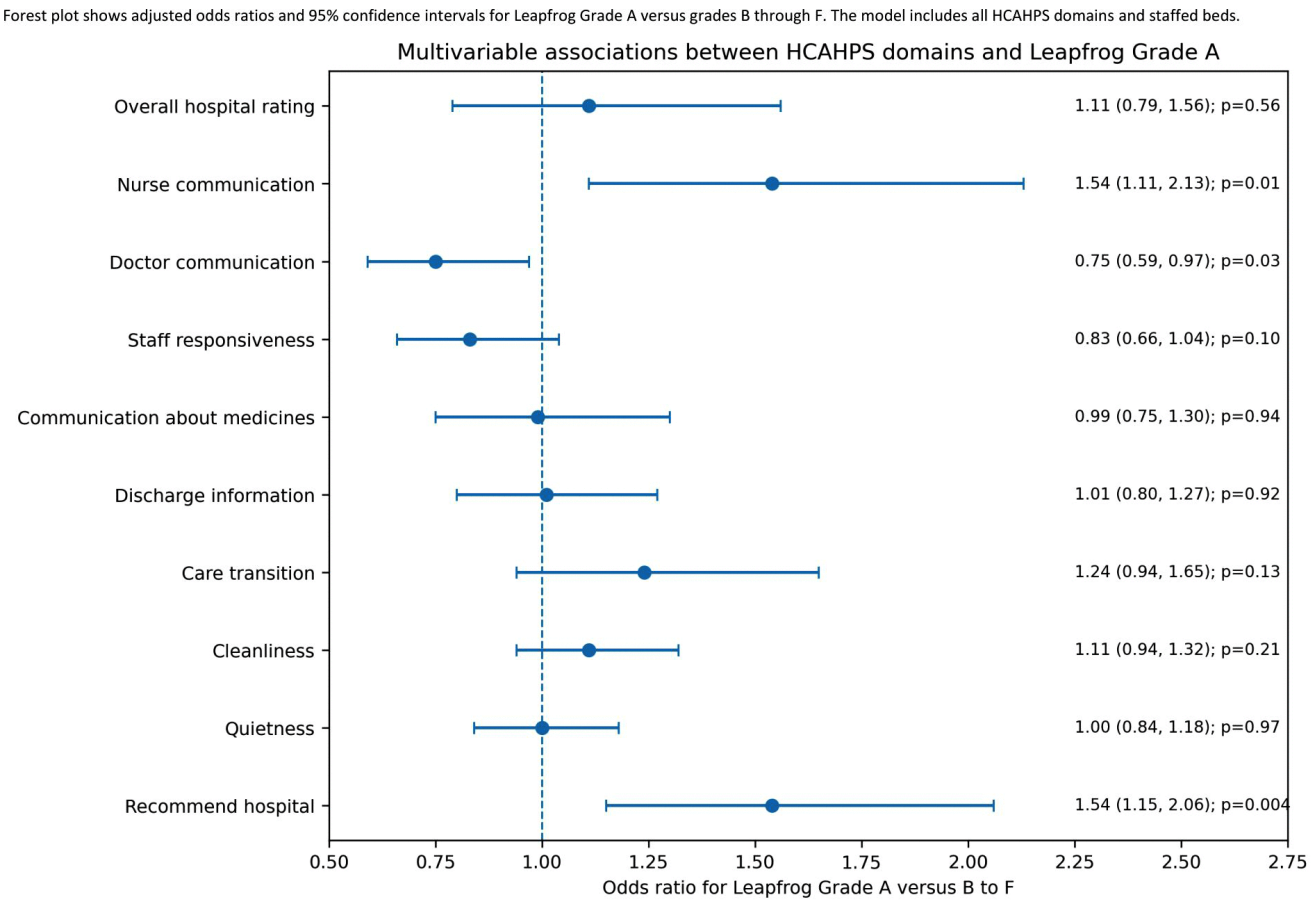
Multivariable associations between HCAHPS domains and Leapfrog Grade A. Forest plot shows odds ratios and 95% confidence intervals for Leapfrog Grade A versus grades B through F. The model included all HCAHPS domains and staffed beds.

In the multivariable logistic regression model, nurse communication was associated with higher odds of achieving Leapfrog Grade A (OR 1.54, 95% CI 1.11 to 2.13; p = 0.01), as was recommend hospital rating (OR 1.54, 95% CI 1.15 to 2.06; p = 0.004) (Table 3; Fig 3). Doctor communication had an inverse conditional association with Grade A status (OR 0.75, 95% CI 0.59 to 0.97; p = 0.03). Overall hospital rating, staff responsiveness, communication about medicines, discharge information, care transition, cleanliness, and quietness were not independently associated with Grade A status after adjustment. Staffed beds had a statistically significant but very small association with Grade A status (OR 1.001, 95% CI 1.000 to 1.001; p = 0.03), suggesting limited practical importance.

**Table 3:** Multivariable logistic regression model predicting Leapfrog Grade A.

Independent variable	Beta coefficient	Odds ratio (95% CI)	p value
Overall hospital rating	0.1006	1.11 (0.79 to 1.56)	0.56
Nurse communication	0.4318	1.54 (1.11 to 2.13)	0.01
Doctor communication	−0.2832	0.75 (0.59 to 0.97)	0.03
Staff responsiveness	−0.1886	0.83 (0.66 to 1.04)	0.10
Communication about medicines	−0.0109	0.99 (0.75 to 1.3)	0.94
Discharge information	0.0119	1.01 (0.8 to 1.27)	0.92
Care transition	0.2167	1.24 (0.94 to 1.65)	0.13
Cleanliness	0.1082	1.11 (0.94 to 1.32)	0.21
Quietness	−0.0037	1 (0.84 to 1.18)	0.97
Recommend hospital	0.4293	1.54 (1.15 to 2.06)	0.004
Staffed beds	0.00077	1 (1–1)	0.03

Model included all HCAHPS domains, staffed beds, and state-fixed effects. Grade A was coded as 1. Leapfrog grades B through F were coded as 0. HCAHPS domains were analyzed on a 1–5 scale, with higher values indicating more favorable patient experience.

## Discussion

In this national hospital level analysis, higher Leapfrog Hospital Safety Grades were generally accompanied by more favorable HCAHPS patient experience scores. The strongest descriptive gradients were observed across overall hospital rating, nurse communication, discharge information, care transition, cleanliness, and recommend hospital scores. In the adjusted model, nurse communication and recommend hospital rating were the most clinically interpretable HCAHPS domains associated with Grade A safety status. These findings support the concept that patient experience and technical safety performance are not isolated constructs; instead, they may reflect overlapping features of hospital culture, communication, reliability, and organizational performance.

Our findings are consistent with prior work suggesting that patient experience is linked to clinical safety and effectiveness, although the relationship is neither universal nor causal [5–7]. National surgical studies have similarly reported associations between patient satisfaction and some objective quality indicators, including mortality, failure to rescue, process performance, and resource use [8–10]. The present analysis extends this literature by examining multiple HCAHPS domains against Leapfrog Hospital Safety Grades in a national sample.

Nurse communication emerged as an important predictor of Grade A safety status. This finding is clinically plausible because nurses are the most continuous bedside interface between patients and the hospital system. Nursing communication may reflect not only interpersonal skill but also staffing, care coordination, medication explanation, discharge preparation, and responsiveness to patient concerns. Prior HCAHPS focused literature has identified communication as a recurrent driver of patient satisfaction, and nursing communication frameworks emphasize listening, empathy, clarity, and individualized interaction as central elements of effective care [15,16].

The inverse adjusted association between doctor communication and Grade A status deserves caution. This result should not be interpreted as evidence that poorer physician communication improves hospital safety. Prior literature generally supports positive relationships between effective physician communication, adherence, satisfaction, and health outcomes [17,18]. A more likely explanation is both statistical and contextual. HCAHPS domains are highly correlated, and when multiple overlapping patient experience domains are entered into the same model, coefficient direction can change because of multicollinearity or suppression effects. In addition, physician communication may be influenced by case complexity, patient expectations, duration and frequency of contact, rounding structures, health literacy mismatch, and hospital workflow. The result is therefore best viewed as a conditional association that requires further study rather than a clinically actionable negative finding.

The broad hospital recommendation score was also independently associated with Grade A. This domain likely integrates multiple dimensions of the patient experience, including perceived communication, trust, coordination, cleanliness, comfort, and confidence in the hospital. Because recommendation scores are global and subjective, they should not replace technical safety measures. However, the association with Grade A status suggests that patients’ overall appraisal of the hospitalization may capture a meaningful signal about organizational performance.

The use of Leapfrog grades as the outcome also requires careful interpretation. The Leapfrog composite safety score was developed to synthesize multiple safety indicators into a consumer facing measure [11]. However, studies comparing hospital rating systems have shown substantial discordance among public rating programs, and Leapfrog related measures may be influenced by reporting structure and voluntary components [12–14]. For this reason, our findings should be interpreted as associations with one prominent safety grade system rather than definitive evidence of overall hospital quality.

From a practical standpoint, the findings suggest that hospitals seeking to improve both patient experience and quality performance should not treat HCAHPS as a customer service exercise alone. Communication, especially nursing communication, may be part of a broader reliability infrastructure that affects patient understanding, escalation of concerns, discharge readiness, and confidence in care. Interventions such as structured interdisciplinary bedside rounding, teaching, empathy training, and standardized discharge communication may be reasonable targets, but future prospective studies are needed to determine whether improving these domains changes safety grade performance or simply reflects hospitals that are already performing well.

Finally, statistical significance should be interpreted alongside clinical importance. In large hospital level datasets, small differences can become statistically significant, especially when domain scores are highly correlated. The staffed bed association in this study, for example, was statistically significant but had little practical interpretability. Similarly, the negative physician communication result should be interpreted in the context of effect size, model structure, and external clinical knowledge [19].

This study has several limitations. First, the cross-sectional, hospital level design precludes causal inference. The results cannot definitively establish that improved patient experience causes better Leapfrog grades or that safety grade performance causes better patient experience. Second, the analysis is ecological and hospital level averages cannot determine whether individual patients who reported better experiences received safer or higher quality care.

Third, the public datasets did not provide sufficient patient level, community level, or hospital structural covariates to adjust for case mix, socioeconomic status, teaching status, ownership, system affiliation, payer mix, local post-acute care access, or neighborhood deprivation. Prior HCAHPS studies have shown that survey mode, patient mix, response rates, and hospital size can influence HCAHPS results [20,21]. Fourth, Leapfrog participation and reporting may introduce selection bias, and Leapfrog grades may not align perfectly with other hospital quality rating systems [12–14]. In addition, although state-level HCAHPS variation was described, the multivariable model did not include state fixed effects; residual geographic confounding may remain. Fifth, the decision to dichotomize Leapfrog Grade A versus all other grades improves interpretability but loses ordinal information. The full grade distribution was therefore reported, and future analyses could consider ordinal or multinomial models if model assumptions and sample sizes are adequate. Sixth, HCAHPS domains are highly correlated, which can make multivariable coefficients sensitive to collinearity and suppressor effects. This is particularly relevant to the inverse doctor communication association. Finally, HCAHPS scores reflect patient reported experience, not direct measures of morbidity, mortality, adverse events, or clinical process adherence. Patient experience is an important component of quality, but it should be interpreted as complementary to, rather than interchangeable with, technical quality and safety measures.

## Conclusions

In this national hospital level analysis, higher Leapfrog safety grades were generally associated with higher patient reported experience scores. In adjusted models, nurse communication and hospital recommendation were the most clinically interpretable HCAHPS domains associated with Leapfrog Grade A status. The inverse conditional association observed for doctor communication should be interpreted cautiously and should not be understood as evidence that physician communication is not important for patient safety. These findings support patient experience as a meaningful but imperfect complement to hospital safety metrics and underscore the need for a comprehensive dataset with benchmarks that rigorously captures both patient safety and satisfaction simultaneously.
